# A patient with acute myocardial infarction with electrocardiogram Aslanger’s pattern

**DOI:** 10.1186/s12872-023-03678-x

**Published:** 2024-01-02

**Authors:** Ming-hao Liu, Hao Li, Ang Li, Ru Liu, Hai-bo Liu, Li-jian Gao, Qing Gu, Lei Song

**Affiliations:** 1 Coronary Heart Disease Center, Department of Cardiology, Fuwai Hospital, CAMS&PUMC. No.167 North Lishi Road, Xicheng District, Beijing, China; 2 People’s Hospital of Bayingoleng Mongolian Autonomous Prefecture, No. 56, Renmin East Road, Korla City, Bayingoleng Mongolian Autonomous Prefecture, Xinjiang Uygur Autonomous Region, China; 3 Interventional Catheterization Laboratory, Fuwai Hospital, CAMS&PUMC, No. 167 North Lishi Road, Xicheng District, Beijing, China; 4 Department of Emergency, Fuwai Hospital, CAMS&PUMC, No. 167 North Lishi Road, Xicheng District, Beijing, China

**Keywords:** Aslanger’s pattern, Acute Myocardial Infarction, ST-segment elevation, Critical coronary stenoses, Revascularization

## Abstract

**Background:**

Aslanger’s pattern in electrocardiogram (ECG) indicates that patients may have acute inferior myocardial infarction(AMI) with concomitant critical stenoses on other coronary arteries, which needs to be evaluated the timing of revascularization as risk equivalents of ST elevation myocardial infarction(STEMI).

**Case Presentation:**

The patient was a 62-year-old male with chief complaint of intermittent exertional subxiphoid pain for 20 days from 30th June. One day after the last episode (19th July), the 18-lead electrocardiogram showed ST segment elevation of 0.05-0.1mV in lead III, ST segment depression in leads I, avL, and V2-V6, T wave inversion with positive terminal vector in lead V4-V5, and positive T wave in lead V6, which indicated Aslanger’s pattern. With increased Troponin I (0.162ng/mL, 0-0.02), The patient was diagnosed as acute non-ST-segment elevation myocardial infarction (NSTEMI) and admitted to coronary ward on 20th July. The coronary angiography showed 95% stenosis in the distal left main coronary artery (LM) to the ostium of the left anterior descending artery (LAD), 90% stenosis in the proximal segment of the LAD, and 80% stenosis in the middle segment of the LAD, and TIMI blood flow was graded score 2. Three drug-eluting stents were implanted at the lesions. The patient’s ECG returned close to normal one month after revascularization.

**Conclusion:**

We presented an acute coronary syndrome case whose ECG showed with Aslanger’s pattern (i.e., isolated ST-segment elevation in lead III, associated ST-segment depression in lead V4-V6 with positive T wave/terminal vector, and greater ST-segment elevation in lead V1 than in lead V2), and was confirmed severe stenosis of the LM and the proximal segment of the LAD via coronary angiography. In clinical practice, especially in the emergency, patients with ECG presenting Aslanger’s pattern should be urgently evaluated with prompt treatment, and the timing of emergency coronary angiography and revascularization should be evaluated to avoid adverse outcomes caused by delayed treatment.

## Background

Aslanger’s pattern [1, 2] was proposed by Aslanger et al. in April 2020 after reviewing 1000 electrograms(ECG) of acute non-ST-segment elevation myocardial infarction (NSTEMI). This ECG pattern includes the following features: (1) Isolated ST-segment elevation in lead III and no ST-segment elevation in the remaining inferior leads; (2) ST segment depression in lead V4-V6 with positive T wave/terminal vector; (3) ST segment elevation in lead V1 was greater than that in lead V2. Aslanger’s pattern usually suggests that patients may have acute inferior myocardial infarction(AMI) with concomitant critical stenoses on other coronary arteries, with larger infarct size and higher mortality. Here we report a case of AMI with ECG Aslanger’s pattern and severe stenosis of the distal left main artery (LM) to proximal left anterior descending artery (LAD) confirmed by coronary angiography.

## Case presentations

**Fig. 1:**
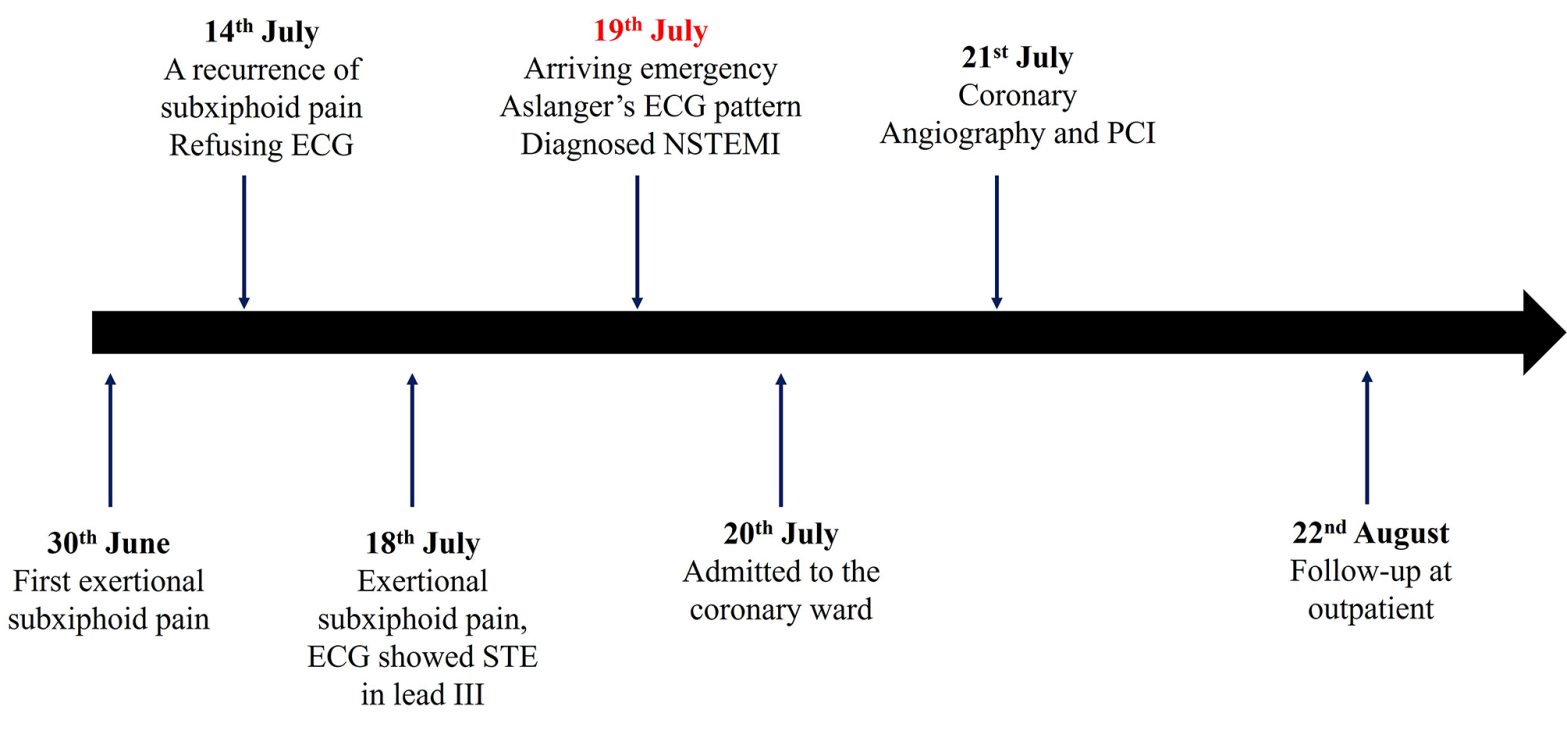
The patient’s timeline from onset of symptoms to treatment. ECG = Electrocardiogram, STE = ST-segment elevation, NSTEMI = Non ST-segment elevation myocardial infarction, PCI = Percutaneous coronary intervention

The patient was a 62-year-old male. His chief complaint was exertional subxiphoid pain for 20 days from 30th June, which lasted about 5–10 min and relieved immediately after cessation of activity with variable frequency. Five days ago (14th July), the patient had a recurrence of subxiphoid pain after breakfast, which lasted for 30–40 min, accompanied by sweating and fatigue. The patient was referred to the emergency of a local hospital, but he refused electrocardiogram (ECG) examination at that time and was relieved by orally taking rabeprazole. One day earlier (18th July), the patient developed exertional subxiphoid pain again, and the emergency electrocardiogram of the local hospital showed “ST segment elevation in lead III”. The patient was treated with “aspirin 100 mg, clopidogrel bisulfate 75 mg, atorvastatin 20 mg” and other drugs. He was referred to our hospital and arrived at our emergency department at 23:30 on 19th July (Fig. 1 Timeline).

The patient had no history of hypertension, diabetes, surgery, food or drug allergy. He had no history of smoking or alcohol use. He had never undergone previous coronary examinations.

**Fig. 2:**
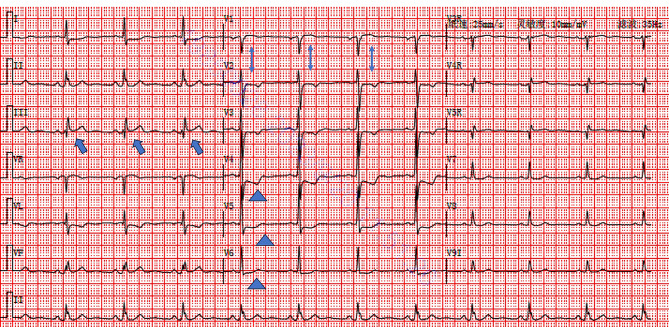
Central Illustration. The patient first electrogram (ECG) in admission in emergency. Bold arrows indicated ST elevation in lead III. Double arrows indicated greater ST segment elevation in lead V1 than V2. Triangles indicated ST segment depression in lead V4-V6 with lead V6 positive T wave and positive terminal vector V4-V5 T wave

Vital signs on admission to the emergency: heart rate 74 beats/min, respiration 20 breaths/min, SpO2 100%, upper limb blood pressure 170/92 mmHg. Physical examination of the heart, lungs, and abdomen showed no obvious abnormalities. The first 18-lead electrocardiogram showed ST segment elevation of 0.05-0.1mV in lead III, ST segment depression in leads I, avL, and V2-V6, T wave inversion with positive terminal vector in lead V4-V5, and positive T wave in lead V6, which indicated Aslanger’s pattern (Fig. 2, Central Illustration). Markers of myocardial injury after admission to the emergency: Troponin I 0.162ng/mL (0-0.02), myoglobin 18.8ng/mL (0-46.6), creatine kinase isoenzyme MB 4.250ng/mL (0-4.99), creatinine 70.98µmol/L (57–111), D-dimer 0.34 mg/L (0-0.55). Echocardiography showed left ventricular end diastolic diameter (LVEDD) 47 mm, left ventricular ejection fraction (LVEF) 63%, and no segmental wall motion abnormality. The patient was diagnosed as acute non-ST-segment elevation myocardial infarction (NSTEMI) due to chest pain, elevated myocardial enzymes, ischemic changes in electrocardiogram, and isolated ST-segment elevation in lead III. Global Registry of Acute Coronary Events (GRACE) score was 126 at admission with intermediate risk.

**Fig. 3:**
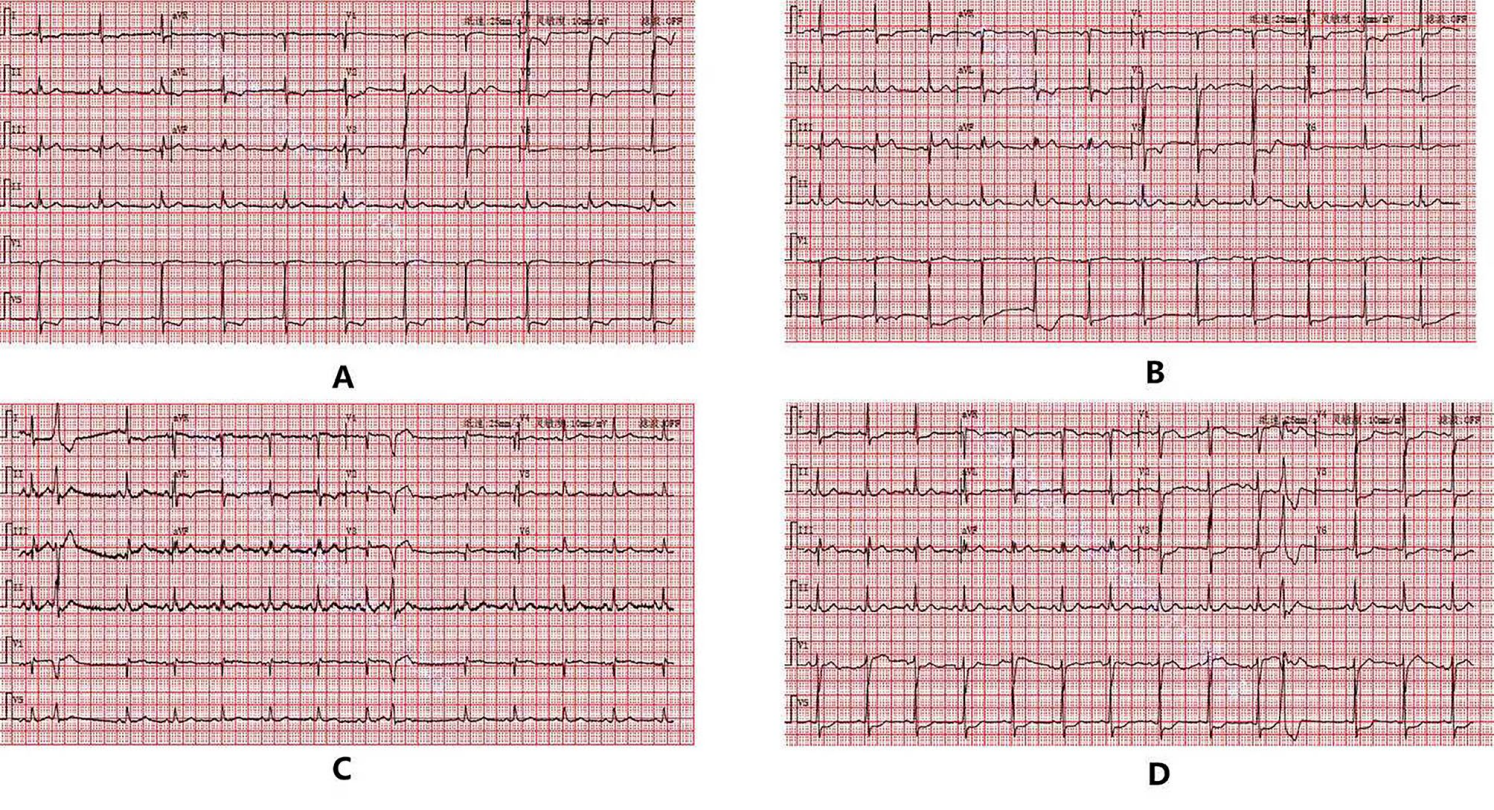
ECGs at different times after admission. **A** was taken when leaving emergency room. **B** was taken when admission in coronary ward. **C** and **D** were taken under his chest pain attack

**Fig. 4:**
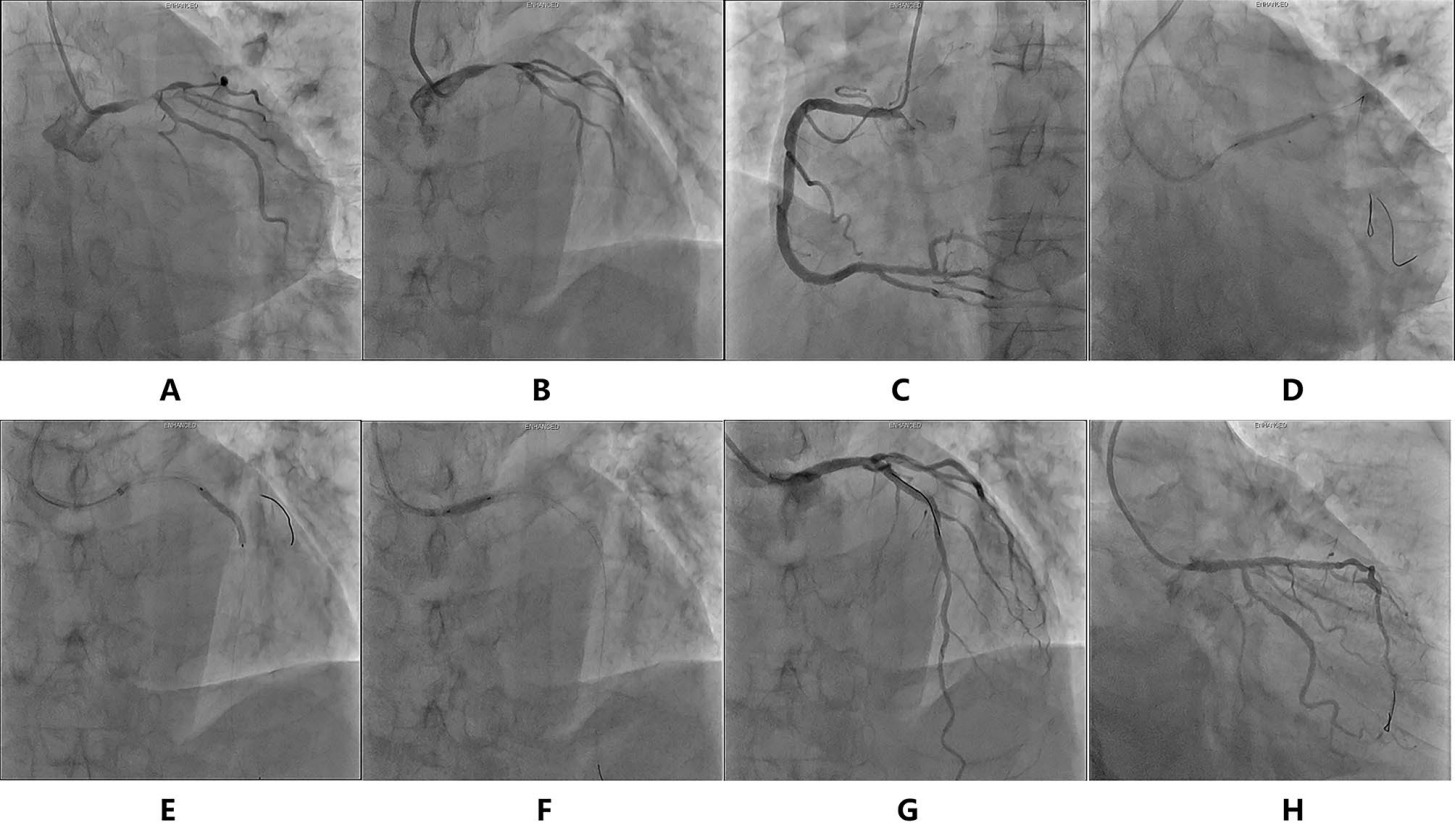
Coronary angiography and intervention. **A-C** indicated severe stenoses of left main artery(LM) and left anterior descending artery(LAD). **D-F** indicated stents insertion in the lesion of LM and LAD. **G-H** were angiography after intervention

The patient was admitted to the coronary ward next day (July 20th ). Further examination showed that his HbA1C was 6.5%, total cholesterol 4.06mmol/L, and LDL-c 2.53mmol/ L, which indicated he has risk factors of diabetes and hypercholesteremia. The patient still had recurrent chest pain during hospitalization, and the T wave and ST segment in the thoracic lead of ECG showed dynamic changes with ventricular premature beats (Fig. 3A-D). On July 21st, the patient underwent coronary angiography (CAG), which showed 95% stenosis in the distal left main coronary artery (LM) to the ostium of the left anterior descending artery (LAD), 90% stenosis in the proximal segment of the LAD, and 80% stenosis in the middle segment of the LAD, and TIMI blood flow was graded score 2 (Fig. 4A-C).

After predilated with The Sprinter 1.5*15 mm balloon and the Quantum Maverick 2.0*15 mm balloon (15-20 atm), a Resolute Integrity 3.0*22 mm drug-eluting stent (DES,Medtronic,USA) was inserted at LM-LAD, a Firebird2 2.5*23 mm DES (MicroPort,China) was implanted distal to the previous stent, and a Tivoli 4.0*10 mm DES (ESSEN,China) were deployed in the left main coronary artery respectively. Left circumflex artery (LCX) was also dilated with balloon using Balloon-stent-kissing technique (Fig. 4D-F). DESs attached and expanded well to the artery confirmed by intravascular ultrasound (IVUS). Final coronary angiography confirmed that the stenosis had been successfully relieved (Fig. 4G-H). After the procedure, tirofiban was intravenously pumped for 6 h, and the antiplatelet regimen was changed to aspirin 75 mg QD and ticagrelor 90 mg BID after discharge. Atorvastatin 20 mg QN, ezetimibe 10 mg QD, captopril 12.5 mg BID, isosorbide mononitrate 20 mg BID were continued to take orally for at least one year. His symptom of chest pain disappeared.

**Fig. 5:**
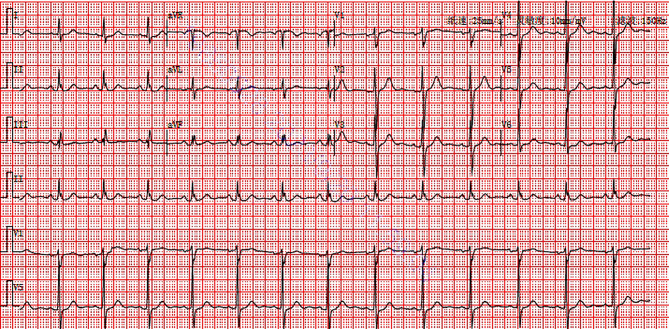
The follow-up ECG one month after operation

One month later (25th August ), the patient returned to the outpatient clinic of our hospital. His blood examination showed total cholesterol 3.16mmol/L, LDL-c 1.41mmol/L, and NT-proBNP 45.0pg/mL, echocardiography showed LVEDD 44 mm, LVEF 71%, electrocardiogram showed low T wave in lead III with ST segment in the isopotential line. The ST segment in the anterior wall lead recovered and the T wave was normal and upright (Fig. 5).

## Discussion

In April 2020, Aslanger et al. reviewed ECG and angiography in 1000 patients with NSTEMI and 1000 patients without MI, and identified patients with acute inferior occlusive myocardial infarction with concomitant critical stenoses on other coronary arteries presenting a specific ECG pattern : (1) Isolated ST-segment elevation in lead III and no ST-segment elevation in the remaining inferior leads; (2) ST segment depression in lead V4-V6 with positive T wave/terminal vector; (3) ST segment elevation in lead V1 was greater than that in lead V2 [1, 2]. In the case of localized inferior-wall injury, the ST vector of inferior myocardial infarction locates the infarct territory and usually points downward and to the right. The ST vector of subendocardial ischemia is not confined to the ischemic territory and points to the aVR lead regardless of which coronary territory is involved. When both vectors were combined and pointed to the right, they caused ST-segment elevation only in leads III and aVR, but ST depression in leads I and II. Since the ischemia vector is perpendicular to the lead avF, the ST segment of the lead avF is always on the isopotential line. Because the ischemia vector is opposite to the lateral wall, the ST segment of the V4-V6 lead is always depressed. The ECGs in our case basically met the above characteristics. Although the typical Aslanger’s pattern does not include ST segment depression in lead V2, ST segment depression in leads V2-V3 can also be seen by the deflection of the ischemia vector when the ischemia range is large. In our case, the patient had severe stenosis of the LM and proximal LAD. The involvement of the LCX may be related to inferior wall ischemia. The stenosis of the LM and proximal LAD caused extensive subendocardial ischemia, and finally the vector pointed to the right, resulting in ST-segment elevation in lead III and ST-segment depression in leads V2-V6.

According to the published data, the ischemia related artery varied in different cases. Zhang et al. reported an elder case with Aslanger’s pattern, in which there was severe stenosis in the distal LM and LAD, and subtotal occlusion of the proximal RCA with visible thrombosis confirmed by CAG [3]. Simon et al. reported another case with Aslanger’s pattern, in which LM and LCX was chosen as first choice to intervene [4]. In our case, the ischemia related lesion was stenosis of distal LM and proximal LAD, which might also influence LCX and manifest as Aslanger’s ECG pattern. The Aslanger’s pattern in ECG was found to occur in 6.3% of NSTEMI patients [1], and it was associated with larger infarct size and higher mortality. The timing of revascularization should be urgently evaluated, and should be even treated as ST-segment elevation myocardial infarction (STEMI) risk equivalents to avoid delaying the timing of revascularization. In this case, we previously diagnosed the patient as NSTEMI with intermediate risk and conduct coronary angiography 2 days after admission. This was an impressive lesson to learn, as the patient did not suffer from severe fatal myocardial infarction or other complications fortunately.

Increasing evidences showed that at least one-fourth acute coronary occlusion (ACO) cases would be misdiagnosed according to current ECG standards of STEMI and its equivalents [5]. Therefore, it is necessary to investigate novel indicators to identify ACO. With the emergence of new ECG indicators [6, 7] and risk scores [8, 9] like Aslanger’s pattern for the identification of acute obstructive myocardial infarction, more ACO cases will be identified early and quickly and given timely revascularization in the future.

## Conclusion

We presented an acute coronary syndrome case whose ECG showed with Aslanger’s pattern (i.e., isolated ST-segment elevation in lead III, associated ST-segment depression in lead V4-V6 with positive T wave/terminal vector, and greater ST-segment elevation in lead V1 than in lead V2), and was confirmed severe stenosis of the LM and the proximal segment of the LAD via coronary angiography. In clinical practice, especially in the emergency, patients with ECG presenting Aslanger’s pattern should be urgently evaluated with prompt treatment, and the timing of emergency coronary angiography and revascularization should be evaluated to avoid adverse outcomes caused by delayed treatment.

## Data Availability

All data supporting the conclusions are presented in the manuscript.

## Declarations

### Perspectives

To understand the relationship between ECG Aslanger’s pattern and severe coronary stenoses and the change of ST segment and vector;

To recognize the ECG Aslanger’s pattern, especially in emergency treatment, coronary angiography and revascularization should be evaluated promptly.

## Abbreviations

ECG: Electrocardiogram
AMI: Acute myocardial infarction
LM: Left main artery
LAD: Left anterior descending artery
LCX: Left circumflex artery
LVEDD: Left ventricular end diastolic diameter
LVEF: Left ventricular ejection fraction
STEMI: ST-segment elevation myocardial infarction
NSTEMI: Non ST-segment elevation myocardial infarction
DES: Drug-eluting stent

## References

[1] Aslanger E, Yıldırımtürk Ö, Şimşek B (2020). A new electrocardiographic pattern indicating inferior Myocardial Infarction. J Electrocardiol.

[2] Miyauchi E, Kuwazuru K, Arikawa R (2023). Clinical features of the Aslanger Pattern to compensate for the limitation of ST-Elevation Myocardial Infarction (STEMI) Criteria. Cureus.

[3] Zhang Q, Zhao Y, Huang X (2022). Acute inferior occlusion Myocardial Infarction with a solitary ST-elevation in lead III: a case report. J Electrocardiol.

[4] Simon A (2023). A proposal for better visualization of Aslanger pattern. J Electrocardiol.

[5] Aslanger EK, Meyers HP, Smith SW (2021). Recognizing electrocardiographically subtle occlusion Myocardial Infarction and differentiating it from mimics: ten steps to or away from cath lab. Turk Kardiyol Dern Ars.

[6] Hayıroğlu Mİ, Lakhani I, Tse G, Çınar T, Çinier G, Tekkeşin Aİ (2020). In-Hospital Prognostic Value of Electrocardiographic Parameters Other Than ST-Segment changes in Acute Myocardial Infarction: literature review and future perspectives. Heart Lung Circ.

[7] Li Z, Chen K, Li S, Liu T. Expert Consensus on ECG Identification Applied in the Insurance Industry. Cardiovasc Innov Appl 2023;8(1).

[8] Hayıroğlu Mİ, Çınar T, Çiçek V (2021). A simple formula to predict echocardiographic diastolic dysfunction-electrocardiographic diastolic index. Herz.

[9] Hayıroğlu Mİ, Çınar T, Selçuk M (2021). The significance of the morphology-voltage-P-wave duration (MVP) ECG score for prediction of in-hospital and long-term atrial fibrillation in ischemic Stroke. J Electrocardiol.

